# Dressing and Addressing the Mental Patient: The Uses of Clothing in the Admission, Care and Employment of Residents in English Provincial Mental Hospitals, c. 1860–1960

**DOI:** 10.1179/0040496914Z.00000000045

**Published:** 2014-11-06

**Authors:** Nicole Baur, Joseph Melling

## Abstract

Scholars of insanity and its historical antecedents have paid very little attention to personal and institutional clothing. Such dress, distributed to patients in mental institutions, has always been inscribed with the conflicting narratives of the period in which it was made and worn. The language of civil and medical authority is more evident than personal choice in the shape and address of the attire. This article examines clothing worn by patients in three Devon mental hospitals during the century before 1960. We consider the ways in which institutional clothing formed part of a hospital regimen of overt control, as well as suiting considerations of economy and employment that figured in these institutions.

## Introduction: Dress, Institutional Design and the History of Mental Illness

**FIG. 1.**
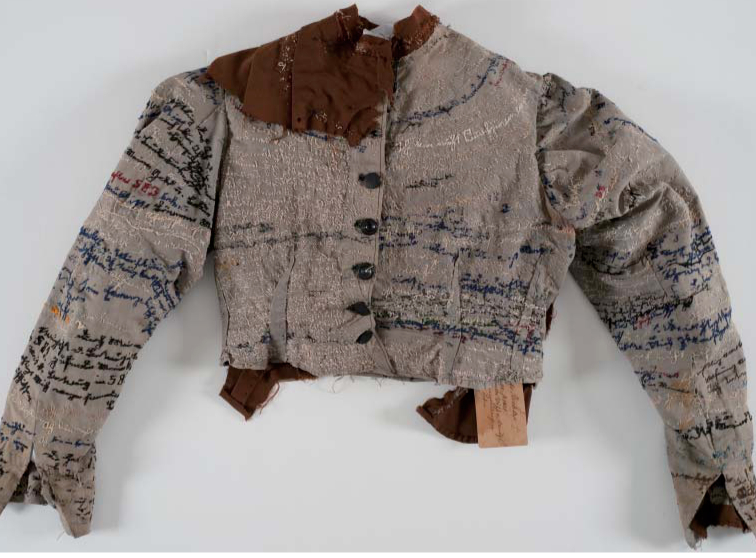
Handmade little jacket, embroidered with autobiographical text, Agnes Emma Richter (1844–1918), Inv. No. 743, c. 1894, thread on hospital linen. ©*Prinzhorn Collection, Centre for Psychosocial Medicine, University Hospital Heidelberg.*

One of the prized exhibits in Heidelberg University Museum is an odd-looking linen jacket dating from about 1895 (Fig. 1). It belonged to Agnes Richter (1844–1918), a mental patient who, diagnosed with *dementia praecox*, spent twenty-five years of her life in a Saxony mental institution. Skilled as a seamstress, Richter remade the apparel issued to her upon admission, stitching it to fit her slender figure. She also painstakingly embroidered personal details across the face of the garment, offering a visual testament to her life story.

Written in German Gothic script, the writing emblazoned but also disguised her narrative, some of which was threaded on the inner lining of her garment. Difficult to decipher, the embroidered script apparently offered Richter an opportunity to ‘talk back’, using her needle to trace out a voice that re-fabricated an identity issued to patients by the institution. The asylum laundry number 583 was given to her (doubling as an identifier for patients) and this figured repeatedly in a ‘tapestry’ of sewn language. This oddly coded form enabled the wearer of the jacket to (it seems) use the surface of her apparel to project, but also to protect, her inner thoughts from the watchful eyes of the institution.^1^

This article suggests that, usually in less vivid ways than in the Richter case, clothing distributed to patients was inscribed with the conflicting narratives of the period in which it was made and worn. The language of civil and medical authority is more evident than personal choice in the shape and address of the attire, although we may also detect different institutional aspirations, as well as the activities of the patients imprinted, sometimes violently, on the garments they wore.

Amid the intense scholarly debate on insanity and its historical antecedents, there has been very little discussion of personal and institutional clothing. Scholars frequently complain that few fragments of patient testimony survive from decades and even centuries of institutional care, whether in the form of diaries and correspondence or personal possessions. It is also evident that surviving items of patients’ clothing are rare relics from this lost world. This is a remarkable absence, for the experience of insanity and mental illness was not confined to a few outcast members of society. Across Europe, North America and the rest of the world millions of patient admissions were recorded by asylums and mental hospitals in the century before the 1960s. Institutional and personal clothing employed thousands of people in and beyond these places even in the late twentieth century. At that period began a great movement for de-institutionalisation of such patients and, by the end of the century, most people suffering from a mental illness were treated outside the large hospitals that had been dedicated to their care in the previous century. Photographs which survive from the distant and recent past suggest the importance of dress in defining the place and role of people within the mental hospital. It is this little-researched aspect of care and habitation that we address here.

Our limited understanding of the role of dress within the closed institution of the mental hospital contrasts with the wider and growing interest of social historians in clothes and their place in the material culture of societies and organisations. Clothes have figured prominently in the history of the economic and physical wellbeing of populations, as well as in the fabrication of societal identities. The making and wearing of clothes has occupied a prominent place in the economic and social history of industrialisation, playing a leading part in the making of modern consumer societies in Europe and elsewhere. Margot Finn has noted the contribution of the consumption of clothes to the making and maintenance of an emotional and sentimental economy in the modern world, in addition to the adornment of polite society by desirable objects.^2^ Pierre Bourdieu and others have argued that the exchange of such gifts deepens the scope of social relationships, extending networks of trust and affection that bind together friends as well as kinship groups in admiration of such material and symbolic investments.^3^ Historians of gender and sexuality have similarly stressed the importance of clothing to social identity, while studies of dress have illustrated the importance of the specific political as well as cultural concerns which surrounded episodes of dress reform and cross-gender dressing.^4^

**FIG. 2.**
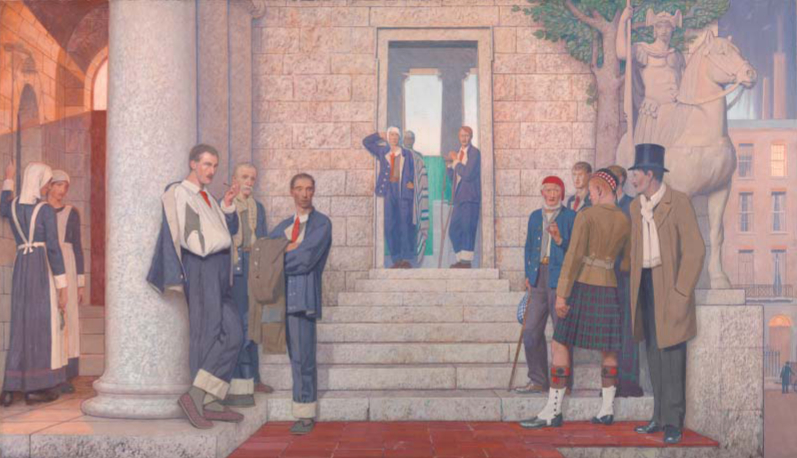
Cayley Robinson, ‘The doctor’: left painting of two. Wounded and sick men gathered outside a hospital, 1920. *Reproduced courtesy of Wellcome Library, London.*

Historians have noted the dangers posed by the manufacture and labour of cloth-making and the hazards of toxic substances used in making and cleaning garments during the past two centuries.^5^ However, discussions of clothing and health have usually dealt with the hazards attendant on manufacture, or the dangers of infection from clothes and the parasites that live upon them, rather than their use within medical institutions.^6^ The role of clothing in regard to the health and well-being of people and the design and development of hospital dress has attracted some interest, gender historians exploring the ways in which clothing figured in the transformation and reorientation of gendered and sexual identities in times of war.^7^ Jeffrey Reznick has discussed in some depth the distinctive standard blue uniform and the armbands, neckties and lapel badges issued to convalescing military patients in hospitals during the Great War (Fig. 2).^8^

In the aftermath of that conflict, the consumption of clothing in European societies reflected the relaxation of attitudes to social propriety and decorum, including sexual expectations of gender. The rejection of ‘repressive’ clothing and the assertion of the right of nakedness have figured in research on the growth of physical culture and gender identities in European societies during the twentieth century, including the place of clothes (and the naked body) in the rise of Communist and Fascist movements which celebrated the robust human physique.^9^

Our understanding of the role of clothing in contemporary perceptions of mental illness remains limited, although dress is frequently alluded to in accounts of insanity. The few popular images that survive often depict agitated patients tied into straitjackets or bound with heavy corsets as relics of an earlier and supposedly less caring era. Even here, the use of restraining garments and the seclusion of patients in padded cells (textile-clad walls forming another feature of ‘clothing’ for the isolated individual), has attracted limited research in recent social history.^10^ This appears a significant omission. An abundance of contemporary evidence indicates the importance of dress to the identity and mental condition of patients in mental hospitals during the nineteenth and twentieth centuries. For if the history of western fashion has been saturated by concerns for sexual allure and encoded with the evolution of gendered identities, so we find that the florid accounts of mental derangement in modern consumer societies are entangled with images of the destructive potential of erotic excess vividly expressed in descriptions of distressed dress.

The decorum or disarrangement of dress provided contemporaries with important clues as to the mental state of an individual. When Millicent B. was admitted to Wonford House Asylum near Exeter on 19 July 1873, she was depicted as an unmarried ‘gentlewoman’ aged thirty, who was ‘bewildered and deluded’. The evidence provided laid particular emphasis on her appearance, her skin being ‘filthy’ and ‘her dress neglected. She had three stockings on’.^11^ Female dress in particular frequently formed part of the portraiture of the insane patient provided in medical notes, drawn from initial certification of insanity and from the reflections of asylum staff following the entry of women into the institution. This appears to have been particularly relevant to descriptions of women who entered fee-paying, as distinct from Poor Law asylums (public assistance institutions funded by local tax payers) during the nineteenth century. Annie B. arrived at Wonford House in November 1884, after spending several years at Bethlem Hospital and another institution. The diagnosis seemed well established, although again her appearance and conduct were significant features in the recorded description of her condition:

On admission she appears to have been erotic, destructive and careless of her dress, but very fond of music and fancy work. Her erotic and destructive tendencies appear to have lessened, but in 1883 she is reported to be full of delusions, among which the principal is that she is Christ.^12^

It was said that Annie had come to the Exeter institution after residing at the seaside during the summer and disturbing onlookers by promenading with her New Testament, including personal diagrams of New Jerusalem, proclaiming herself as Christ. It was reported that her destructive tendencies had extended to her clothing.^13^ Different shades of interpretation about the mental condition of this unmarried female were woven around her dress, singling out the ways in which her religious delusions as well as her erotic and destructive tendencies were expressed in her attire and demeanour, scandalising the public.

The clothes of men and the wilful destruction of their dress attracted less attention in the medical accounts of insanity in English provincial asylums during the nineteenth century than those of women, although the dirty and dishevelled appearance of male lunatics (as well as their physical health and appearance) was often commented upon. The loss of male reason was more likely to be explained in relation to individuals’ incapacity to fulfil their masculine duties in employment and public life, and by evident misconduct or violent assault. The disarray of dress in men could point to mishaps in personal conduct and public demeanour, although the re-imposition of correct dress codes was generally seen as less important to the redress of lapses in mental and moral balance than in the case of women, as we shall show in the remainder of this article.

The purpose of this article is to open a scholarly discussion on the changing role of clothing in the recognition and treatment of insanity in England during the century after 1860. Interpreting the significance of clothing in the social and institutional lives of people found to be insane and mentally ill remains a challenging task. For clothing may have signified a state of normality, but the terms in which ‘normal’ clothing was understood depended on both expert and popular opinion, subject to cultural change during the decades we examine. These may refer to clothes or utilise the imagery of clothing as a way of explaining patients presenting a persona, such as descriptions of ‘flamboyant’ or ‘extravagant’ dress and behaviour. One recent American study suggests that certain schizophrenic patients wear ‘redundant clothing’ although the authors do not develop a historical analysis of their findings.^14^ Clothing may also signal esteem among carers. A recent study reported that nurses admitted that they ‘look upon patients differently, depending upon if they wear personal or institutional clothing’.^15^ The nature and quality of garments can influence the status of people inside, as well as outside, medical institutions. We could say that patients’ clothing was one way in which the institution dressed itself, contributing to an interior landscape perceived by members of the hospital community and also defining a nexus between that community and the wider world.

Psychiatrists and social scientists have provided commentaries on patients at different points in the history of mental illness and these suggest one starting point for an examination of the role of clothing in the contemporary recognition of sanity and insanity. In a celebrated discussion of schizophrenia, first published in 1964, R. D. Laing noted that people with an insecure knowledge of the world and their place in it may seek to camouflage themselves, even ‘disappearing’ from the living world. Clothing figured in some of the relationships and quarrels recorded between patients suffering from schizophrenia and their parents.^16^ Laing reported that one of his female patients believed that a child had been killed wearing her clothes, murdered either by herself or her mother.^17^

The most influential account of institutional life in medical facilities developed at the end of our period was provided by the sociologist Erving Goffman, whose studies of hospitals revealed the different ways in which people came to understand themselves and to participate in the regimes of control and regulation that exercised many critics of mental health care during the 1950s and 1960s. Goffman showed how such regimes of regulation served to depersonalise and even stigmatise patients, separating them effectively from the outside world. Clothing given to patients acted to camouflage their personal identity, transforming them from autonomous individuals into recognised and recognisable patients, whose separate personalities became merged with (and were appropriated by) the institution. The removal of personal possessions, including clothing, can be said to have stripped the sense of self from patients on entering the institution.^18^

The reforming psychiatrist Russell Barton documented the impact of such loss of personal possessions on patients in the late 1950s, the dispossession of the patient and the lack of space in which to store personal belongings, contributing to what he termed ‘institutional neurosis’:

Large numbers of patients in some mental hospitals have no place in which they can keep personal possessions, no lockers by their bed. Keeping all patients’ possessions in a single property room is a bad system. It means in practice the property is hardly ever available for the patient to use. Often clothes are issued to a ward and there may be no guarantee that if a patient keeps her frock clean one day she will wear the same on the next day. Similarly photographs of her family, writing paper and such essentials as combs, toothbrushes, cosmetics, etc., are difficult and often impossible to keep.^19^

Barton emphasised that among the features contributing to the ‘ward atmosphere’ were the design of rooms and ‘rugs, carpets, cushions, curtains’, although he stressed the collective as well as individual impact of patients’ appearance, including ‘hair styles, hair on faces, clothes, stockings, shoes’. For Barton, the impression given by the dress and demeanour of the human community, as well as the arrangement of physical objects, were vital to patient outlook, for drab surroundings communicated ‘the idea that “nothing matters” which fosters the apathy being produced by other pressures’.^20^Similar challenges faced people who suffered, or were said to suffer, from various forms of ‘mental deficiency’ (later termed ‘learning difficulties’), where pyjamas could figure in determined struggles by patients to assert control over their choice of day clothes.^21^

At different periods in the nineteenth and twentieth centuries, observers drew insights from the incidence of insanity in society to offer a vision of social relations, sometimes using metaphors of clothing in discussions of belonging and social place. Historians are required to be sensitive to contemporary usages in language and ideas, as well as changing fashions in scientific practice and popular taste in dress. Scientific and sociological accounts of clothing cannot by themselves adequately encompass historical change in the function and meaning of institutional garments. At any point in time one object may signal relationships as much as usage. Function depends on context and recognition. The critical, reformist writing of the 1950s and 1960s, including the project of ‘anti- psychiatry’, supposedly promoted by a counter-culture of libertarian writers and historians from Goffman to Laing and Thomas Szasz to Andrew Scull, has been itself subject to revisionist challenges.^22^

To analyse mental hospitals this article draws on social, scientific as well as historical explanations to propose that we should see clothes worn inside an institution as speaking about more than historical identity: we argue that they also signified societal and institutional presence. Clothing demarcated and described to a significant degree the personal space which enabled individuals to develop and defend their identification of the self. In this respect personal dress expressed the individual and the person’s relationship to objects. This relationship betokened more than rights to individual property, although the persona of the patient was necessarily shaped by social background and by contemporary customary expectations. Differences in class and status were registered in the demands of more affluent patients for greater recognition of the needs and desires of the self, even within the confines of mental illness. Within mental institutions, people might defend their personal identity and sense of self by the continued possession of particular objects, including clothing, over time spent inside the institution’s boundaries, while the use of jackets and corsets to restrain mental patients indicates one way in which garments were used to control recalcitrant or non-compliant individuals physically. These expectations and capacities changed over time, as various moves to liberalise tightly regulated regimes were periodically made during the century after 1860.

These changes within the prescribed order of English mental hospitals arose not only from intellectual persuasion but from the agency of patients and relatives as well as staff and hospital governors. To understand the rhythm of relationships between the different agents, we may conceive clothing as forming part of the transactions conducted with, and within, the institutional space that all patients and their carers occupied. Clothes also connected the person with a past and future self. The night shirt or night gown may be simply bed attire, but if patients are so dressed during the day then they are being held in a particular relationship to different objects and purposes. Their ability to enter spaces wearing such dress provokes considerations of appropriate space as well as approved times, as we saw in the cases of the women admitted to Wonford House in the nineteenth century, mentioned above.

The rules governing the dress of patients and of the staff who attended them also altered over time. The dressing of people in establishment ‘uniforms’ may not always have been designed to signal authority and discipline over those who were so clothed, although in some instances at least clothing could perform a punitive role. For the most part, garments as well as institutional life followed a regular routine, overseen by an external inspectorate (under Lunacy legislation passed in 1845), who ensured basic standards were respected. For example, the Lunacy Commissioners noted of the Devon County Lunatic Asylum at Exminster during a visit in the mid-nineteenth century that the ‘bedding and clothing of the patients were clean, neat, and of good quality’, with patients ‘fairly satisfactorily clothed’.^23^

As we noted in the case of Agnes Richter, the voice of the patients is less easily retrieved than official and medical opinion, although fragmentary documentation of their experience of mental illness includes descriptions of dress to express deeper concerns, such revelations being sometimes recorded by medical attendants. Nevertheless, recovering the history of patient clothing remains difficult. That which was so visible and readily recognisable to contemporaries may be strangely hidden from historians. With the exception of photographs inserted on early patient files, hospital records reveal little as to what kind of garments the patients were actually wearing. Occasional references in reports of visits by the Lunacy Commission provide snapshots of patient dress from which we may stitch a narrative of clothing, although the meanings attached to this apparel has to be uncovered from a range of textual and oral sources. Utilising this diverse range of possible evidence to understand the part played by clothing in the history of mental wellbeing requires, therefore, some awareness of changing views of illness as well as of contemporary understanding of dress in the identification of roles and authority in different institutional settings. This article contributes to the subject by an examination of the admission, treatment and discharge of patients from provincial mental hospitals in Devon during the century after 1860.^24^

## Coming to the Asylum: The Place of Clothing in the Admission of Patients

Our research into clothing worn by mental patients in the century before 1960 was designed to discover the extent to which institutional clothing formed part of a hospital regimen of overt control, as well as meeting considerations of economy and employment which figured in the discussions of some institutions.^25^

Distinctive clothes were certainly designed, made and issued for different work tasks undertaken by inmates in Devon hospitals, such as cleaning, washing, farming, gardening, laundering and scrubbing, resembling in some respects the hard-wearing apparel familiar to labouring people outside the gates of the institution. Only patients considered to be at particular risk of harm or suspected of intent to escape appear to have been required to wear distinctive uniforms, most residents being issued even in public asylums with clothing that differed little from everyday wear seen in local neighbourhoods. In fee-paying establishments there was even less requirement to don institutional dress. The emphasis was rather on using disciplined routines, increasing privileges and personal apparel to remind inmates of their previous and appropriate station in life and of the behaviour expected of them as a means of recovering their reason. This was vividly apparent in the treatment of groups such as governesses, who had been responsible for the instruction of others in social decorum before they lost their sense and sensibility.^26^ Even in these respects, we may say that the more liberal regimen of the institution remained emblazoned on the apparel of the inmates and on the garments of those attending and directing them. This article argues that institutional clothing provided by hospitals should be seen as part of the journey of patients into and through the institution to which they were committed. This involves attempting to recapture the significance of clothing at distinct points in the journey into and out of the asylum and mental hospital. We suggest that clothing figured as a significant facet in the making and management of patients over these years.

The penal and punitive features of the regime were formed within a regulatory order which sought to meet competing objectives and needs. The institutional culture of these places was permeated with patterns of privilege and tolerance, registering internal and external demands for the care of people with different conditions and unequal resources. The practical care of those admitted to the asylum fell on the shoulders of attendants who were more likely to have been trained in the military, police force or in domestic service than educated in medical duties. The tiny medical staff and their workforce of attendants were given the task of containing, controlling and correcting aberrant behaviour and delusions of individuals while ensuring that a large and growing population of inmates was not disrupted beyond the boundaries of order. These boundaries were equated with the realm of reason and authority, to which the disordered minds were expected to conform. The most common diagnoses of both male and female patients throughout the nineteenth century was ‘mania’ (including excessive egotism or ‘monomania’), followed by melancholia and dementia.^27^

Dress played a notable part in sustaining this enterprise. Clothes also reflected contemporary views of the patients and the most appropriate means of dealing with their disease. The state of the dress of individuals in different settings was frequently offered as a reasonable basis for suspicion, or even evidence of insanity during the century after the passage of major lunacy reforms in the 1840s. Comments on clothing figured in the admission documents of a variety of individuals at different periods. Expectations of correct attire were connected to ideas about the proper conduct of the body. The soiling of clothes was taken as a potent sign of an individual’s incapacity to care for themselves and an inability, or determined unwillingness, to control their bodily functions. ‘Dirty habits’ appeared frequently in descriptions of patients and ranged from a cursory comment on the incontinence of the demented or deranged to a condemnation of a collapse in physical and moral standards. The failure to dress hair and to shave bodily hair, as well as coverage of the body, could register the presence of mental derangement for the observer who was composing a portrait of insanity. In other instances it was not a neglect of bodily economy but a deplorable failure to attend to the requisites of household management and necessary thrift which betrayed the feckless lunatic, for relatives would report the purchase of clothes that were not required and consequent indebtedness. Inside the institution, the mere requirement of frequent washing and bathing could call forth the disapproving description of ‘dirty’, although the poorly paid asylum staff of Victorian times were certainly hard pressed to deal with increases in their duties and the necessity of replacing bedding as well as clothing.^28^

The prevalence of ‘dirty habits’ also ensured that the more difficult, obstreperous or simply helpless inmates of the local Poor Law ‘lunatic ward’ (which survived in many workhouses later than the 1870s when reforms were introduced to Poor Law workhouse provision) were transferred to the county and borough asylums built in such large numbers from the 1840s. The destruction of clothes and bedding, as well as threatening behaviour, persuaded many Poor Law Guardians of the need for transport to the asylum. In this sense, clothes featured as one visible passport of the dirty and disruptive individual to the asylum, but also diminished the prospects of a high cure rate being recorded by the asylum authorities and contributed to the growing pessimism that clouded discussions of insanity during the last three decades of the nineteenth century. Many, mostly elderly, patients were sent to hospital having failed to clothe themselves adequately in different seasons or having shown visible signs of negligence or disregard of dress. One patient was described as having ‘set out in the middle of the morning without breakfast, in clothes that did not match and with [her] hair streaming loose’.^29^Leaving female hair unpinned seemed almost as threatening to sane arrangements as leaving a garment carelessly unbuttoned. For proper clothing betokened a respect for decorum as much as property and probity. An eccentric relationship with clothing, such as a refusal to wear a truss or undress at night, could frequently be taken as a sign of mental deterioration.

Even more shocking to contemporaries than the besmirching or rending of garments was their absence. The spectacle of nudity or even partial undress usually secured arrest and often a rapid entry to an asylum during this period. The language of nakedness was again deployed in a variety of ways according to the requirements of documentary evidence and the purpose of the author. When the first patients were admitted to the Devon County Lunatic Asylum in 1845, the ‘nude’ condition of female arrivals was harshly criticised by the Medical Superintendent Charles Bucknill when a female was brought by Poor Law officers entirely naked.^30^ Admissions included a man found bathing naked in a local fountain, and such displays of bodily parts continued to warrant police attention during the twentieth century.^31^ Public and indecent exposure, sometimes coupled with allegations of assault, led the culprit to the magistrates and thence to the asylum if the latter, acting in their capacity as the Visitors or legal governors of the Victorian asylum, decided on a committal order to these Poor Law institutions. Although Devon did not possess a criminal or forensic psychiatric service in our period, a number of patients were admitted to the Devon hospitals after exposing themselves or committing indecent assault — frequently directly from police custody. Dangerous patients were usually despatched to Broadmoor and Rampton, only the less dangerous remaining after committing sexual misdemeanours, including the theft and/or misuse of female underwear and other items of clothing from washing lines across the county. A number would return to prison once their period of treatment was concluded. In discussions of the dangerous and the criminal, references to ‘dirty habits’ are frequently difficult to interpret since they may denote incontinence, wilful fouling of clothes and bedding, or moral misdemeanours — such as masturbation.^32^

The language of dirt was not restricted to a description of filthy, soiled or ragged clothing, but could also indicate a material or emotional threat to the institution. Different items of clothing might indeed have presented some kind of risk to the patient population of mental hospitals such as those in Devon, with ragged garments often seen as a danger to health by infection or lice. On their admission to the Devon County Lunatic Asylum at Exminster (built near Exeter in 1845 but serving the whole of Devon), patients undressed for a full physical examination. Details of bodily condition and features, as well as mental attitude were noted in admission registers and in some cases photographs were taken. Patients were then given a hot bath, as distinct from cold baths for those who were unruly or disturbed after a period of residence, and issued with a standard hospital dress for wear inside the wards and the grounds of the asylum.^33^ The little evidence we have suggests patients’ clothing was placed in storage during their residence, which on average lasted between two and five years for those who were to be discharged. Access to personal and hospital clothing was clearly restricted and a close watch was kept on the clothing of patients thought to be suicidal or capable of harm. After bathing, patients were assigned a bed in one of the wards, with unruly admissions kept in nightclothes during the first days of their stay at the asylum, under the watchful eye of attendants. This restriction in the clothing of the individual was only one of the ways in which garments functioned to underwrite the authority of the medical staff and we can now consider in more depth the regime which developed at these institutions.

## Dressing the Institution: Clothing Lunatic Asylum Patients

**FIG. 3.**
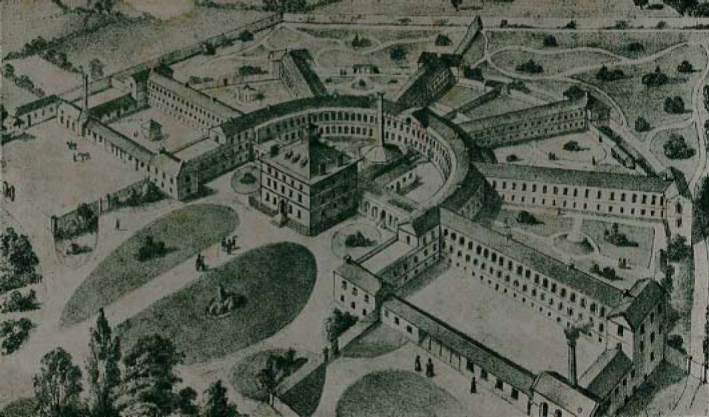
The Devon County Lunatic Asylum, 1845. *Reproduced courtesy of Devon Heritage Centre, Exeter.*

The architects of the large county asylums of the nineteenth century presented a vision of pastoral serenity, surrounding the massive central blocks of buildings with parklands and recreational lawns that often gave unbroken views to distant hills and estuaries (Fig. 3).

**FIG. 4.**
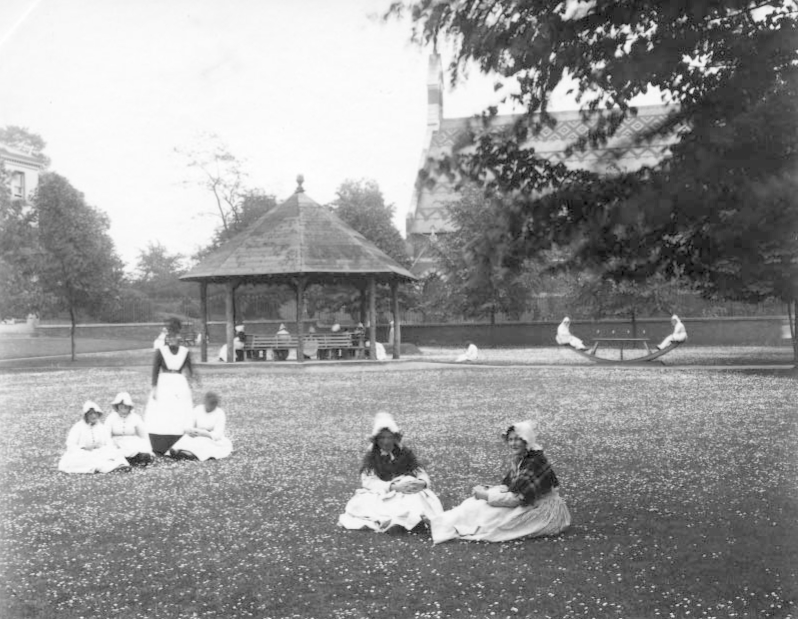
Patients clothed in institutional dress at Horton Road Asylum, Gloucestershire, c. 1890s. *Reproduced courtesy of Gloucestershire Archives, reference HO22/27/3.*

The interior of such establishments was also designed to encourage respectable conduct and orderly communion in galleries and airing courts. These daily routines preserved the decorum and discipline of gender as well as authority, one former Devon Mental Hospital (the Devon County Lunatic Asylum was renamed Devon Mental Hospital in 1930) employee recalling that, even in the twentieth century, ‘the male wards looked a bit austere — like a barrack room — but sparkling clean. The female wards looked more homely, very pretty with their little tablecloths and flowers and the sisters in their white starched uniforms’.^34^ The concern of medical staff and attendants was to integrate the individual into this world as quickly as possible. Once admitted to the asylum, the patients were issued with standard clean clothing, rather than marked garments or uniforms (Fig. 4). This served to provide poorer or destitute individuals with adequate garments, although letters of complaint sent by relatives to the Devon County Lunatic Asylum Superintendent in the early decades of the twentieth century indicate that more affluent members of the working population saw the removal of personal garments as an affront to their family dignity. Relatives also wrote to the medical staff suggesting that the wearing of personal clothes would speed the recovery of their family member.^35^

There is little to suggest that the garments offered to new admissions were shoddy. The quality of the clothing was good enough to tempt staff to pilfer some items for personal use or sale. The Devon County Lunatic Asylum Superintendent, George Saunders, reported in 1871 that seven of the male attendants had ‘been using and wearing the Asylum clothing, and that there was reason to suspect that they had carried away some of the stores’. Warrants were issued to search their houses and one of the attendants was prosecuted.^36^ Whether attendants appropriated the clothes for use or wore them as disguise when stealing from the stores is unclear.

The maintenance of clothes in good order also continued to be taken by asylum staff as an indication of the progress of the individual within the ordered routine of the institution. Unruly residents were made to remain in their night clothes during the day, on the principle that they could be more easily managed. Even in relatively recent times some patients who presented little threat to the nursing regime have been dressed in night gowns because they were ‘prone to wander out of the hospital given the slightest opportunity and could not be reasoned with owing to [their] dementia’.^37^ The removal or restriction of clothing from recalcitrant inmates were the subject of criticism as early as the 1870s, *The Lancet* objecting on ethical grounds to the common practice of confining patients who had destroyed their clothes to a single room or cell, even leaving them naked and without bedding for considerable periods.^38^ Remarks about clothing featured in patient case notes, including references to ‘slovenly’ habits in dress. Dressing later than the hospital routine required was also commented upon. A patient might be dressed on the specific order of the institution’s Medical Officer, whether they had been previously in nightwear or simply failed to dress themselves. One patient, for example, ‘dressed after lunch’.^39^ Less frequent were positive comments about the patients’ action of dressing, for example after an illness, which was seen as a sign of recovery.^40^

The clothes issued by institutions were intended to integrate patients into a shared identity, perhaps limiting conflict over personal possessions and signs of different social status. Absconding patients were more easily recognised, even if it was too late to detain them, for any inmate remaining free for two weeks could only be readmitted on fresh certification. One patient who escaped from the Devon County Lunatic Asylum in ward clothing was found some six months later hanging from a tree and identified by his asylum clothes, although it was unclear if he had remained in these garments during his freedom or donned them for his death.^41^ The use of clothing, as well as bedding or window cords, for suicide was a constant concern and belts were removed from patients on suicide watch. The regulation of dress, the preservation of health and the disciplining of asylum inmates were so frequently meshed together in accounts of institutional life that it is misleading to imply a clear separation of motives within the rationale of the encompassing regime.

To assess the safety of patients admitted to asylums and mental hospitals we need to compare repeated public statements that the ‘bedding and clothing of the Patients were neat, clean and of good quality’,^42^ with the evidence that in the early years of the Devon County Lunatic Asylum mattresses were simply stuffed with straw and the first upholsterer only appointed in 1893.^43^ Patients were sometimes tied, quite literally, to their beds to preserve order, while the place of bedding and tagged or numbered night attire remained a tool in the identification of patients that survived many legislation reforms in the nineteenth and early twentieth century. Yet it was not the condition of their clothing or bedding that posed the most significant threat to patient health in our period, but rather the persistent and cumulative problems of overcrowding within the walls of the institution. At the best of times, the great asylums offered little more than rest cures for the majority of people who passed through their doors. This posed particular problems where chronic and infectious diseases such as tuberculosis could be spread so effectively. A sanatorium with disinfecting chamber was opened at the Devon County Lunatic Asylum in 1877, but the facility was ineffective and reassigned in the face of an urgent need to relieve overcrowding by housing male patients, becoming functional only in 1923.^44^ It was the lack of personal and communal space rather than dirt which presented the greatest challenges and most lethal threat to the health of residents.

In such overcrowded conditions the space and tolerance allowed to disruptive patients was almost inevitably limited. The new asylums of the 1840s were constructed on a wave of optimism that insanity could be cured, primarily without resorting to the physical restraints and punishments that had been discredited in scandals which preceded the age of reform.^45^ Restraints were not forbidden but legislation required that their use be recorded. The most distinctive form of asylum clothing was that designed to restrain refractory individuals, preventing them from harming themselves and others. The Lunacy Act of 1890, superseding that of 1845, made specific reference to such garments, including straitjackets buttoned at the back and fingerless gloves. Such clothing could be worn on successive days for up to ten or eleven hours where considered necessary, including where patients had to be restrained from undressing their own surgical binding for wounds or infection. The Devon County Lunatic Asylum used long-sleeved jackets and gloves on patients who were ‘excited and violent, maniacal and restless’.^46^ The Mental Treatment Act of 1930 was passed in an era where such restraints were discouraged, the central Board of Control (which replaced Lunacy Commissioners before 1914) noting in 1935 that it was ‘interesting to learn that no strong clothing has been required on the male side for twenty years’.^47^ Seclusion of patients had been used from the early days of the Devon County Lunatic Asylum and continued after 1930 (when it was renamed became the Devon Mental Hospital), with opiates also being used on more restive and dangerous patients.

Before considering changes which took place in mental hospital clothing during the twentieth century, it is worth recalling that the making, repair and cleaning of clothes provided a major source of employment for patients as well as staff from the early days of these institutions. A significant number of male and particularly female entrants to the Devon asylums had been employed in some aspect of the textiles, dressmaking and tailoring trades before their arrival. The presence of gloveresses and lace makers reflected the existence of strong specialist employment in north and east Devon respectively, while laundresses were employed across the county.^48^ Such skills were useful to asylums such as the Devon County Lunatic Asylum, where the making of garments, boots and bedding was carried out in the later nineteenth century. Men were also engaged in tailoring, shoemaking, mat making and laundry work from 1848 as well as agricultural and handicraft work outside, while women were more likely to be confined to housework and kitchen duties, although some worked as sewers, knitters and washers. While work was generally regarded in Victorian times as therapeutic and a necessary condition of normal life, the systematic employment of people on tasks for their rehabilitative value was not understood until Dr Simon’s work at Gütersloh, Germany, in the early twentieth century. The Devon County Lunatic Asylum was to embrace it enthusiastically, and by the 1930s enjoyed a national reputation for its scheme of occupational therapy.^49^

Although less skilled and more arduous, laundry work was vital to the internal economy of an institution such as the Devon County Lunatic Asylum, where eight thousand articles were washed each week at the end of the nineteenth century. Most hands were female, although male patients undertook the heavy manual work of mangle-turning and working the wringing and washing machines. Women actually washed the clothes. Mechanisation did not necessarily mean lighter work, although fewer numbers of inmates continued to carry a mass of washing and repairing work throughout much of the twentieth century. In 1945 the laundry needs of almost 1,500 patients were met by only thirteen paid employees and a large group of patients. A few years later the Devon Mental Hospital’s laundry handled twenty-seven thousand articles per week, with a complement of eighty-one workers, sixty-five of whom were patients.^50^

While Digby Asylum (the Exeter City institution noted earlier) laundered only a quarter of this number, the merging of the three Devon hospitals in the 1960s prompted the centralisation of Devon Mental Hospital facilities at the Exminster site of the original County Asylum. A sign of the changing times was the expression of criticism of these arrangements and inefficiencies in distribution. These comments highlighted the fact that patients only began to have their garments tagged with their name after the First World War and shortages or lost items remained a problem later, even in the era of the National Health Service from 1948. Patients remained part of an enforced collective of issued and laundered apparel without control over the return even of the most personal underwear. Devon was not alone in the drab and shapeless quality of some of its clothing or the paucity of its facilities, as John Hopton’s study of Prestwich in the 1930s reveals, with underwear being shared among the patients until the very end of our period.^51^

These bleak examples and rising complaints signal that twentieth-century reforms to strengthen patient rights and dignity advanced, at best, unevenly. Care is needed when constructing a chronology of institutional provision, for the pattern was neither one of unyielding immiseration nor of unbroken improvements. The final section of the article evaluates the nature and pace of innovation after the Victorian era.

## From Asylum Inmate to Mental Patient: The Changing Appearance of Institutional Care during the Twentieth Century

**FIG. 5.**
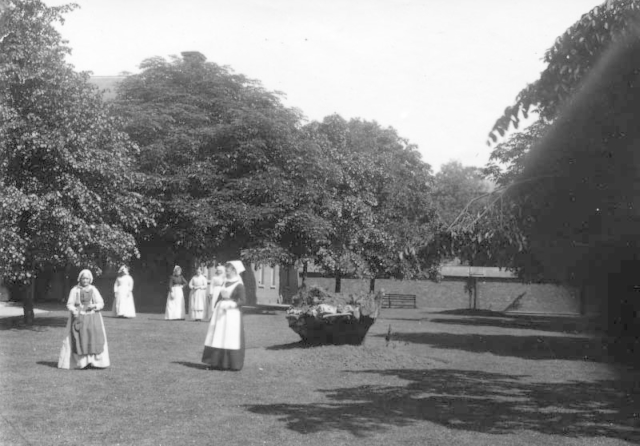
Female patient (in foreground) clothed in garments suitable for employment, Horton Road Asylum, Gloucestershire, c. 1890s. *Reproduced courtesy of Gloucestershire Archives, reference HO22/27/3.*

In the early years of the twentieth century it was common practice to photograph patients on admission to hospital in Devon. These and other images show patients clothed in plain, serviceable garments made of material such as strong linen that was difficult to damage and destroy (Fig. 5).

By the 1950s there had been a noticeable change in the style of dress and the scope which patients were allowed to vary their appearance and to make choices about their clothing. We can trace in the apparel of the patients a visible liberalisation of the regulatory regime which governed their lives in hospital, although the forces behind these changes were varied and frequently in conflict with the concerns of those managing the institutions. The capacity and inclination of the medical directors to relax the hospital regime and improve facilities for patients were circumscribed by a driving concern for economy as hospital costs escalated after the local government legislation of the 1880s passed responsibility for these establishments from magistrates to county and borough councils.^52^ These large medical establishments remained a burden on Poor Law and local authority ratepayers and such institutions competed for fee-paying and Poor Law patients from other areas as a means of achieving more income, gaining economies of scale and reducing their overall costs. One certain way of attracting such clients was to offer a lower rate of maintenance for pauper patients than rival institutions, and this necessarily required economy in clothing and other items of expenditure.^53^ The financial accounts of Digby and Exminster (‘Mental Hospitals’ from 1930) indicate that the cost of clothing rose significantly in the early decades of the twentieth century and continued to do so after the introduction of the National Health Service in 1948.^54^ Local ratepayers had shown a decided hostility to increases in Poor Law rates to fund asylum paupers in the mid-nineteenth century and resistance to rising expenditure remained a feature of local politics into the twentieth.

Such conservatism was countered by three related movements which worked to transform institutional life in the decades after 1914. These were, firstly, legislative reform and the demands of external inspectors; secondly, changes in medical opinion in regard to mental health and therapies for its relief and cure; thirdly, a growing diversity in the social background and expectations of those admitted to public, state-funded, mental hospitals. During the inter-war years the legal and medical authorities in charge of these establishments faced conflicting pressures: they were charged with clothing and housing a large and shifting population within limited budgets which were still formally those of Poor Law institutions, while facing growing demands for improvements in such matters as patient dress and diet. One turning point was the passage of the Mental Treatment Act of 1930 which formally designated all asylums as ‘mental hospitals’ and introduced new categories of Voluntary and Temporary patients in addition to those certified for compulsory detention. The reform movement which led to the fresh legislation also contributed to an awareness of a need to minimise rather than accentuate the differences between the hospital regime and appearances in the outside world. The Board of Control noted of Digby in 1935:

The patients on both sides were tidily dressed, and though we are aware that as in other hospitals there is still a stock of the older type of women’s dresses we are glad to find that garments of a more modern style and more in conformity with those outside are being introduced.^55^

Such efforts to modernise clothes and to cut them in accordance with contemporary fashion reflected a growing recognition that institutional dress de-personalised patients. The femininity of female clothing appears to have been seen as a means of promoting recovery, rather than an invitation to sexual liberation.

The trend in harmonising garments was confirmed by legislative authority but there had been moves to relax the strict dress code of the mental institution before the 1930s. By the early 1920s Exeter’s City Asylum at Digby was providing male night shirt as well as female dressing gowns and efforts were made to remove institutional uniformity and permit personal association with clothing, the Board of Control being pleased to find that the dresses of the female patients were all ‘marked with the names of the wearers and were of nice patterns and materials’. They recommended that the same system be applied to footwear, ‘which would ensure patients having their own boots’.^56^

By the inter-war years, clothes lists had been introduced, detailing the apparel and footwear available for patients performing different tasks at different periods of the year, with seasonal adjustments made, although the way such lists were compiled and the number of items issued to different individuals remains unclear.^57^ The liberalisation in hospital dress was due, in part at least, to changes in medical opinion and to the pressure brought to bear by campaigning journalism in professional organs such as *The Lancet* and *The British Medical Journal*. In 1922 *The Lancet* criticised asylum clothing, calling for greater ‘individuality and normality’ in apparel, including an encouragement to patients to retain their own clothes when in hospital.^58^ The same journal commended the greater freedom given to fee-paying mental patients to create a ‘homely atmosphere’ within the walls of the institution.^59^

Even in Victorian times fee-paying asylums had been more attentive to the wishes of patients and relatives than might be expected, allowing individuals such as Millicent B. to wear their own clothes, viewing the recovery of the appearance as well as the manners of polite society as a necessary feature in the rehabilitation of their ‘guests’. Such patients brought with them personal items of jewellery, watches, manicure sets, rosaries, coat hangers, towels, brushes, mirror and combs, clothes brushes, tie pins, as well as modesty vests, corsets, boudoir caps, glove stretchers and even their own bedding, dining sets and recreational equipment.^60^ The Devon County Lunatic Asylum admitted relatively few fee-paying patients, although there was growing pressure on all establishments to cater for such admissions as one means of balancing the hospital accounts and from its inception as a City asylum, Digby had a dedicated set of preferential rooms and beds for non-Exeter pauper patients and those paying private fees. The numbers of such patients increased markedly during the inter-war years, more particularly after the introduction of Voluntary and Temporary admissions in 1930, when more middle-class patients were seen in public mental hospitals.^61^

**FIG. 6.**
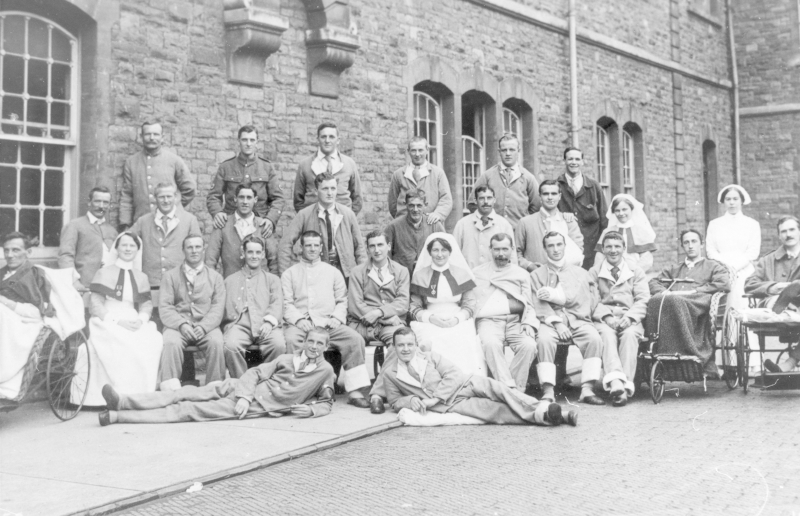
Service patients and medical staff at Beaufort Hospital, Stapleton, undated. *Reproduced courtesy of Glenside Museum, Bristol.*

The contribution of one group of patients to the clothing regimes of the mental hospital seems to have been particularly notable, although it has been largely forgotten. These were military personnel who were admitted as Service Patients with a mental illness after 1914. The Victorian asylums had always housed a number of men from the armed forces and the impact of the Great War on the understanding of male trauma has been well documented.^62^ The presence of patients in receipt of an award from the Ministry of Pensions inside the mental hospitals has attracted less attention, although we know that cancer victims who had been on military service were also among those in receipt of such pensions.^63^ These former servicemen were visited by a dedicated Pensions Ministry official and their clothing allowance was substantial for the period (Fig. 6). The Ministry Inspector commented in 1927 of the Digby Asylum patients that they were ‘neat and clean in person and dress, each man having two lounge suits and personal underclothing of good quality; all are provided with overcoats, nightshirts and toothbrushes’.^64^

The emphasis appears to have been on promoting and preserving a ‘clean, neat and well-dressed appearance’, with complete outfits and formal collars and ties, all provided ‘for personal wear’, reflecting well on their attendants as well as the institution.^65^

The cumulative impact of the increased diversity in the patient population during the inter-war years appears to have been to strengthen the spread of therapeutic treatments which were intended to diminish the stigma and segregation experienced by mental patients and ensured their reintegration into wider society. Efforts to harmonise and improve the clothing of Devon admissions continued after the Second World War. In 1945 the Devon Mental Hospital at Exminster allowed patients to wear their personal clothing and in 1952 Digby Hospital was commended by the Board of Control when they observed that ‘the clothing of both male and female patients appear to us to be well studied’.^66^ We know little about the design of such clothing or the extent to which the Devon hospitals issued varied dress even in the post-war years, although we may assume that many of the long-stay patients, who had been frequently registered as hopeless ‘chronic’ cases in earlier times, were the most likely to have been clothed in hospital garments.

We suggested above that the dress code of an institution was bound up with its regulation of space as well as time, and that the designation and command of personal space is integral to the recognition of personal identity. Changes in rules that governed the clothing of patients may have had little impact on the patient’s sense of self, if access to those garments and discretion over the selection of items for wear remained in the hands of others. Restrictions on the resources given to mental hospitals and the management of budgets by medical and administrative staff could lead inexorably to an institutional logic which denied personal space as impractical rather than undesirable. Large central establishments designed and conducted for the majority of people from a wide geographical area were faced with the periodic but serious challenges of overcrowding as they catered for long-term as well as shorter-term patients. The original architecture of Victorian buildings, with long shared dormitories and corridors, and the periodic overcrowding of the buildings at the Devon County Lunatic Asylum in particular, meant that private space and storage remained a problem into the mid-twentieth century. Pressures intensified during both world wars as the hospitals were commandeered for military as well as civilian use. Emergency treatment for soldiers compelled fee- paying patients to share space with those maintained on the rates, again prompting complaints, while the patients’ clothing and food ration books were handed over to the hospital administrators for the period of their stay.^67^

Shortage of storage space had become a long-standing issue by the inter-war years. The loss of personal items, from garments to jewellery and dentures, prompted angry letters from relatives.^68^ The Board of Control described conditions in 1938 at the Devon Mental Hospital, congratulating the staff on the continuing ‘excellent standard, both in quality and variety’ of clothing for female and male patients. Their criticism, which foreshadowed Barton’s later comments (quoted earlier), was reserved for the storage amenities, noting that some patients only had use of a single drawer to store clothes and private possessions:

The lack of storage space for patients’ day clothes during the night was particularly noticeable in some of the female wards, and we are glad to hear that additional coat hangers are being made to provide a partial solution to this difficulty.^69^

The hanging of clothes exercised staff as well as inspectors, offending one deputy matron’s sense of order and decency, as she recalled her dismay in discovering ‘the patients had nowhere to hang their clothes’.^70^ By 1949 an ‘experimental clothes rack’ was being provided in a female ward at Digby Hospital, one witness recalling that this was ‘obviously an improvement on storing clothes in bundles and it appears to be popular. That would have been a great innovation at that time’.^71^ By the late 1960s the three hospital sites had been brought under one management as Exe Vale Hospital and patients were now allowed their own clothes, providing they were ‘adequately marked’ and their possessions listed. Restrictions still applied to suicidal patients. Those without adequate clothing were supplied with any articles of ‘clothing, or combs, toothbrushes, tooth powder, soap, and some shoe cleaning, if necessary’.^72^

Finally, it is worth noting that the hospital year was also marked by moments of celebration and commemoration at which different kinds of clothing were worn. Victorian commentators (including Charles Dickens) had described formal gatherings and events, including the holding of balls and musical entertainments, where patients were invited to take part in a formal social occasion. Staff and selected patients would play musical instruments to an audience that included the Medical Superintendent, his colleagues and invited guests as well as the residents of the institution.^73^ As late as the 1930s the local press would report on the enjoyment patients and staff alike had at the annual fancy dress balls.^74^ It would be stretching the evidence too far to suggest that such events served a similar purpose to the medieval ‘carnival of fools’, where those in authority exchanged places with the lower orders for a public ritual, although the considerable investment of time in such gatherings indicates that the place of clothing in the ritual of recognition and amusement was not lost on those who arranged the dressing up of patients for the establishment’s big occasions.

## Conclusions: Uncovering and Recovering the Closed Institution

Those who observed insanity usually provided ‘facts’ that described the appearance and the behaviour of those suspected of being unsound of mind. Admission documents from the nineteenth century and later frequently detailed loose clothing and uncombed hair as evidence of the lack of personal care and bodily control by an individual. An unbuttoned garment betrayed incompetence in the presentation of the self and threatened the loss of social regard. Social and cultural historians are now alert to the significance of clothing in the making and preservation of personal identity and as a register of changes in societal relations of class, gender and age. It is therefore surprising that the clothing of those diagnosed as insane has attracted so little attention in the history of textiles. This article has argued that this is an important omission. Dress was not only a significant feature in the detection, description and alleviation of insanity, but the subject of clothing figured notably in discussions of the regulation and control of hospital populations between the 1860s and the 1960s.

Critical surveys of large mental hospitals undertaken in the 1950s and 1960s often presented a bleak, unchanging world of conformity and discipline which defined virtually every aspect of institutional life. These accounts transformed contemporary understanding of the ways that institutions constrained the capacity of individuals to express themselves and to assert a distinct identity within the fabric of a regulatory regime. Such regimes were often driven by concerns beyond those of personal expression or even medical care. However, the critical assault on the large mental hospital also obscured some of the significant changes which altered patient experiences in the century we have surveyed. There is now a more nuanced and careful assessment of the human costs and benefits of such establishments, although it remains clear that patients were seen primarily as members of a closed community directed by those in authority, without the need for the active consent of residents. As the machinery of the great laundry system indicates, individual identity and possessions were seen for many years as simply impractical and concessions such as patient lockers as an unnecessary gift of personal space. Only gradually was choice recognised as an instrument of recovery.

This article suggests that late nineteenth- and early twentieth-century hospitals were not ‘total’ institutions which sealed off their occupants from the outside world, locking them into warehouses of long wards where no effective treatment was offered. Innovations as well as constraints originated beyond the walls of the asylum. To understand the origins and the scope of such reforms, we have argued that clothing should be understood in transactional terms, as one of the means by which people defined the relationship between the institution, its residents and wider society. Relatives as well as magistrates, doctors, journalists, Poor Law Guardians, staff associations, county councillors, philanthropists and others provided a running commentary on the affairs of these establishments and their proper relation to the society they served. Such interactions were not simply a matter of common sense or consent: they often represented struggles for rights and the intrusion of progressive opinion from without.

The portraits of the patients, from the photographs of the early twentieth century to the accounts of concerned relations, provide a narrative on the routes by which these people were dressed as well as addressed. Admissions to mental institutions reveal the way in which many patients signalled a descent into mental illness through the neglect, soiling or destruction of clothing. What form of inner disquiet was displayed by this prominent distressing of cloth is often unclear, although the regulated world of the asylum placed a heavy emphasis on uniformity of outward clothes and a discouragement of any personal excesses in either appearance or in the ‘derangement’ of garments. The concern with conformity and the reduction of variation, as well as individual caprice, among the patient population may also have provided some reassuring basic standard available to the poorest inmates. Hospital clothing may have ensured some crude equality of dress as well as underlining an enforced collectivity of the patient community. Asylum clothing was made of robust material and was sufficiently durable to survive any encounters with physical strength as well as seasonal climate. External pressure as well as internal initiatives led to a shift in thinking about clothes, ranging from a means of distinguishing the good order of the institution from the outside lives of its patients, to a concern by the early twentieth century with maintaining social links and even peculiar identities, through to dress as a means of recovering the health of the individual.

A concern with good order and economy persisted over the period, with clothing forming one weapon in the battery of mechanical restraint that the mental hospital could use when behavioural sanctions, seclusion and opiates failed to quell the violent or recalcitrant patient. Studies of modern hospitals have noted more subtle, but similar, means of punishment and exclusion by restricting patients to nightwear. The dangers of suicide and escape, both of which were subject to close external scrutiny by the Board of Control, clearly exercised the staff in their regulation of clothing in this period. The majority of patients were not violent, nor were they dirty and destructive in their habits when at the hospital. Most were not admitted for their sexual interest in clothing or for uncovering their bodies. Patients still followed a regulatory regime where garments were issued to patients and all items despatched to a central laundry — from whence personal items might never be returned. In most cases name tags were an invention of the mid-twentieth century. Many patients may even have been involved in the making, repair and cleaning of the thousands of objects that flowed through these large establishments each week.

On several occasions the different Devon mental hospitals we have studied were warmly commended for their dress sense, including the bright patterns of female clothing and their tolerance of individual choice in dress. One solvent of the old order of uniformity may have come from the housing of Service patients whose wardrobe, funded by the Pensions Ministry, was generous by comparison with Poor Law patients. The Mental Treatment Act and introduction of Voluntary patients, including many individuals whose work involved the wearing of white collars and ties, may also have contributed to the reform movement in hospital dress. By the 1920s there were calls for female and male patients not only to have more fashionable garments to wear, bringing them closer to the standards seen outside the walls of the institution, but also to have clothes and even shoes tagged. The fundamental challenge, we suggest, was not to allow a variety of clothes or an association with ‘owners’, but to create a space that could be claimed within the regulatory structure of the institution. Here the older dormitory-driven order of the asylum lived on within the original architecture of the hospitals, reinforced by the dense overcrowding to which many establishments were subjected in the later nineteenth and twentieth century. Only gradually were facilities introduced for clothes to be hung, rather than folded, while personal lockers or storage cupboards were a noticeably late reform.

We can trace a significant move in hospital policy from the enforced collective wearing of similar dress to monitoring individual choice in clothing within mental institutions during the century before 1960. There remained clear restrictions on what was considered ‘practical’ even after 1945. Recognising the agency of patients and of other groups within and without the walls of these establishments should not obscure the formidable continuities in dress as well as design that meant patients often experienced long, mundane wards and corridors filled with people dressed in standard clothes that offered limited prospects for personal expression and the recovery of a sense of self. The future development of large mental hospitals was only seriously questioned after the passage of the 1959 Mental Health Act and in the 1960s many hospital building complexes still possessed the great laundries which remained as working monuments to the key place of clothing in the life of these Victorian edifices.

